# Obsessive‐Compulsive Symptoms Associated With Bupropion Treatment: A Case Series

**DOI:** 10.1155/crps/8097103

**Published:** 2026-06-11

**Authors:** Jose Alfonso Ontiveros y Sánchez de la Barquera, Luis Alberto De La Garza García, Guillermo Sánchez-Torres, Pablo Patricio Zárate Garza, Diego José Vázquez Ramos

**Affiliations:** ^1^ Department of Psychiatry, “Dr Jose Eleuterio Gonzalez” University Hospital, UANL, Monterrey, Nuevo Leon, Mexico, uanl.mx

**Keywords:** bupropion, case report, depression, obsessive behavior

## Abstract

**Background:**

Bupropion is a norepinephrine–dopamine reuptake inhibitor (NDRI) and nicotinic antagonist. It belongs to the class of aminoketones and is commonly used to treat major depressive disorder (MDD) and to aid in smoking cessation. There is scarce literature on the sudden appearance of obsessive‐compulsive symptoms (OCSs) in depressed patients that could be related to bupropion treatment.

**Case Series:**

We present four cases of bupropion associated with OCSs in patients with affective disorders. Patients reported no previous history of obsessive ideation or compulsion. Symptoms appeared after the start of bupropion treatment with a progressive increase in intensity. Upon discontinuation of the drug, there was a notable reduction in symptoms with a complete remission over time. Evaluation of obsessive and compulsive symptoms and evolution were assessed using the Yale–Brown obsessive‐compulsive scale (YBOCS) and clinical global impression–severity (CGI‐S) scale.

**Conclusions:**

The neurobiological basis for the development of OCSs associated with bupropion treatment is not yet fully understood; clinicians need to take these symptoms into account when prescribing bupropion. Further studies with a larger sample of patients with depression who are undergoing bupropion treatment are required to confirm this phenomenon.

## 1. Introduction

Major depressive disorder (MDD) is a common condition worldwide, causing significant distress and presenting a considerable economic burden. It affects a notable percentage of adults, with a prevalence of 8.3% reported among adults in the US [1–4]. Around 65%–70% of individuals with depression experience positive effects and responses to antidepressant medication. However, the remaining individuals fall into the category of treatment‐resistant depression (TRD) [5, 6].

Bupropion is a norepinephrine–dopamine reuptake inhibitor (NDRI) and a nicotinic antagonist, belonging to the class of compounds known as aminoketones [7]. It works by inhibiting dopamine and norepinephrine transporters (DAT and NET), which reduces the reuptake of these neurotransmitters. However, the exact mechanism by which it produces its therapeutic effects is not fully understood [8]. The drug also acts to a lesser degree on nicotinic and serotonin receptors. Bupropion is indicated for the treatment of depression, bipolar depression, the prevention of seasonal depressive disorder, and as a therapeutic measure to quit smoking and add‐on treatment for TRD [9]. Common side effects are nervousness and insomnia. Nausea is less commonly reported in bupropion treatment than SSRI treatment. Bupropion causes the least amount of sexual dysfunction compared to other antidepressants [10].

According to DSM‐5‐TR, obsessive‐compulsive disorder (OCD) is a chronic and often disabling psychiatric condition characterized by the presence of obsessions, compulsions, or both, which are time‐consuming or cause clinically significant distress or functional impairment [11]. Obsessions are defined as recurrent and persistent thoughts, urges, or images that are experienced as intrusive and unwanted, and that typically provoke marked anxiety or distress. Compulsions are repetitive behaviors or mental acts performed in response to obsessions or according to rigid rules, aimed at reducing distress or preventing a feared outcome. According to DSM‐5‐TR criteria, these symptoms must not be attributable to the physiological effects of a substance or another medical condition. OCD is a heterogeneous disorder with multiple clinical subtypes, including contamination/cleaning, checking, symmetry/ordering, taboo thoughts, and relationship‐centered OCD (rOCD), among others. The observations in this report are intended to apply to OCD subtypes, including those that are often overlooked in clinical practice [11, 12].

Research reports on the appearance of obsessive symptoms while using bupropion are very scarce. Vulink et al. [13] reported worsening obsessive thoughts in patients with OCD who were being treated with bupropion. They observed that 8 out of 12 patients showed a 21% increase on the Yale–Brown obsessive‐compulsive scale (YBOCS) score [14], indicating a deterioration of obsessive‐compulsive symptoms (OCSs) [13]. Nevertheless, reports of bupropion on MDD or bipolar depression and the appearance of OCS are scarce.

This report examines four cases: three involving MDD and one involving bipolar depression, all of which had no prior history of OCD and were diagnosed according to DSM‐5 criteria. In these cases, treatment with bupropion resulted in the development of OCS. Remission was achieved after the medication was discontinued. Multiple assessment tools were employed in the clinical evaluation of each case (Table 1). The Y‐BOCS and clinical global impression–severity (CGI‐S) scales were utilized to assess OCSs, thereby creating a longitudinal clinical profile for each case, as shown in Table 2.

**Table 1:** Assessment instruments employed.

Case	Assessment instruments
Case 1	MINI MADRS YBOCS CGI‐S

Case 2	MINI YBOCS CGI‐S

Case 3	MINI Y‐BOCS CGI‐S

Case 4	MINI Y‐BOCS CGI‐S

Abbreviations: CGI‐S, clinical global impression–severity; MADRS, Montgomery–Åsberg depression rating scale; MINI, mini‐international neuropsychiatric interview; YBOCS, Yale–Brown obsessive‐compulsive scale.

**Table 2:** YBOCS and CGI‐S changes after bupropion withdrawal.

Case	Sex	Baseline	4 weeks	8 weeks	16 weeks
Case 1	Male	—	—	—	—
YBOCS obsessions	—	19	8	4	3
YBOCS compulsions	—	0	0	0	0
YBOCS total score	—	19	8	4	3
CGI‐S	—	5	3	2	2

Case 2	Male	—	—	—	—
YBOCS obsessions	—	11	5	0	0
YBOCS compulsions	—	16	8	1	0
YBOCS total score	—	27	13	1	0
CGI‐S	—	6	3	1	1

Case 3	Female	—	—	—	—
YBOCS obsessions	—	16	8	0	0
YBOCS compulsions	—	19	4	0	0
YBOCS total score	—	35	12	0	0
CGI‐S	—	6	4	1	1

Case 4	Female	—	—	—	—
YBOCS obsessions	—	18	10	8	6
YBOCS compulsions	—	0	0	0	0
YBOCS total score	—	18	10	8	6
CGI‐S	—	5	4	4	3

Mean					
Mean YBOCS total score	—	24.7	10.75	3.25	2.25
Mean CGI‐S	—	5.5	3.5	2	1.75

## 2. Case Series Methods

This case series was conducted at a tertiary outpatient psychiatry clinic between Jan 2023–May 2025. Cases were identified prospectively through convenience sampling during routine clinical practice. Eligible patients were those who developed new‐onset OCS during treatment with bupropion for a depressive episode.

Inclusion criteria were: (1) age ≥18 years; (2) diagnosis of MDD or bipolar I disorder with a depressive episode according to DSM‐5 TR criteria; (3) treatment with bupropion; and (4) emergence of clinically significant new‐onset OCS possibly associated with bupropion exposure. Clinically significant OCS was defined as the presence of obsessions and/or compulsions causing distress and functional impairment and a Y‐BOCS total score ≥16 at symptom onset.

Exclusion criteria included: (1) lifetime history of OCD or prior clinically significant OCS (DSM‐5 TR); (2) comorbid psychotic or neurocognitive disorders; and (3) active substance use disorder.

Symptom severity was assessed prospectively using the Y‐BOCS and the CGI‐S scale. All assessments were performed by the same psychiatrist (Jose Alfonso Ontiveros y Sánchez de la Barquera). Baseline assessment was conducted at the time of OCS emergence and identified. Scales were re‐administered 1 month later, with additional assessments at weeks 8 and 16 of follow‐up.

Clinical response was defined as a ≥50% reduction in Y‐BOCS total score from baseline. Remission was defined as a Y‐BOCS total score ≤8. After identification of OCS, bupropion was discontinued in all cases, and patients were followed longitudinally to document symptom evolution and clinical outcomes. This case series was prepared and reported in accordance with the CARE guidelines (Supporting Information).

### 2.1. Ethical Statement

Written informed consent was obtained from the patient for publication of this case report and any accompanying clinical details. All identifying information has been removed to protect patient confidentiality and anonymity. This report was conducted in accordance with the ethical principles outlined in the Declaration of Helsinki. Institutional review board approval was not required according to local institutional policies because this study represents a retrospective case series without experimental intervention.

## 3. Case Presentation

### 3.1. Case 1

A 30‐year‐old single male, businessman presented to the psychiatric outpatient clinic with depressive symptoms involving anhedonia, constant sadness, anxiety, and insomnia. There was no evidence of suicide ideation based on the mini‐international neuropsychiatric interview (MINI) and the Montgomery–Åsberg depression rating scale (MADRS). The patient had a history of dysthymic disorder since the age of 17 and MDD for 22 years, as well as an intermittent history of alcohol and marijuana use. Family psychiatric history showed a father with bipolar II disorder and alcoholism. The diagnosis of the patient was made using the MINI, confirming MDD, dysthymic disorder, alcohol and cannabis abuse and excluding OCD. No relevant medical conditions were detected.

After a thorough clinical examination of symptoms and past interventions (2 previous SSRIs, escitalopram and sertraline with moderate improvement), treatment with duloxetine 60 mg was indicated due to persistent depressive symptoms and complaints of decreased sexual libido with SSRIs. After 8 weeks of treatment, the depressive symptoms persisted with insomnia, anxiety, anhedonia, and excessive guilty feelings. Bupropion 150 mg up to 300 mg was then indicated for TRD and minimized the impact on sexual function. After 4 months of treatment, the patient complained of bruxism with duloxetine, so it was progressively discontinued showing improvement of this side effect. The patient reported after 4 weeks of being only on bupropion treatment a marked improvement in depressive symptoms.

However, after 3 months of being only on bupropion, the emergence of progressive, intense, and intrusive sex thoughts as well as the urge to see female and male genitalia were reported. These symptoms also caused severe distress and anxiety. The patient was aware of the irrational nature of his symptoms but could not avoid them. A reassessment of OCS using DSM‐5 criteria discarded the diagnosis of OCD. Evaluation of OCS was done with YBOCS and CGI‐S scales (Table 2).

Due to the chronological association of the symptoms and the start of the treatment, bupropion was discontinued and fluvoxamine 50 mg up to 150 mg was started. After 4 weeks the patient reported a decrease in OCS with a marked progressive improvement at weeks 8 and 16. Fluvoxamine was suspended at week 8 without showing OCS relapse. The patient reported a remission of depressive symptoms as well OCS after 8 months of observation.

The patient experienced intrusive same‐sex–themed obsessive thoughts and impulses, which he initially perceived as catastrophic and distressing, given his self‐identified heterosexual orientation. Following symptom remission, the patient retrospectively attributed the onset of these intrusive thoughts to their temporal association with bupropion treatment. However, no follow‐up investigations were conducted to confirm or refute this attribution, and the relationship remains observational.

### 3.2. Case 2

A 48‐year‐old single male dedicated to real estate administration, he returned to the outpatient psychiatric clinic due to refractory depressive symptoms (anhedonia, excessive guilt, insomnia, and sadness). The patient has had a psychiatric history of TRD since age 31 (MINI) and Crohn’s disease since age 30, which was in remission for the last 8 years. (actual treatment: mesalazine, prednisone, and azathioprine). The laboratory results indicated a slightly low platelet count. Family psychiatric history includes a mother with a diagnosis of generalized anxiety disorder. His treatment at the time of evaluation included mirtazapine 30 mg, amisulpride 100 mg, and clorazepate 2 mg.

Bupropion was prescribed 150 mg up to 300 mg/day to improve his persistent affective symptoms. Three months later the patient reported disabling OCS, that includes the need to check locks before leaving the house and before going to sleep and checking windows and stoves for fear of having left them open/on for at least 2 h per day. The patient had never felt or reported obsessive ideation or compulsions before. He was in distress and conscious of the illogical nature of the ideas and compulsions but could not avoid them. An anamnesis was performed again in search of diagnosis of OCD with a lack of criteria according to DSM‐5. Evaluation of OCS was done with YBOCS and CGI‐S scales (Table 2).

It was decided to start fluvoxamine 50 mg up to 100 mg to improve OCS with a poor response after 2 months. Therefore, it was decided to continue fluvoxamine and progressively reduce the dose of bupropion. The patient reported a partial but rapid improvement of OCS after 4 weeks with a reduction of time‐consuming compulsions from 2 h to 45 min. A complete remission of OCS symptoms was observed at weeks 8 and 16. Fluvoxamine dose was then reduced to 50 mg and mirtazapine to 15 mg/day due to diurnal somnolence. No return of OCS was observed after 2 years of follow‐up.

The patient reported feeling no longer depressed as a result of treatment. However, he experienced OCS that caused significant anxiety and interfered with his daily functioning. He believed these symptoms were related to his depression. The prognosis for OCS associated with bupropion treatment was considered very favorable.

### 3.3. Case 3

A 35‐year‐old female housewife outpatient, mother of two children with a diagnosis of panic disorder with agoraphobia since the age of 16 and MDD since the age of 28, under long‐term treatment with paroxetine 20 mg, and alprazolam 0.25 mg bid reported daytime sleepiness, weight gain, and decreased libido associated with her treatment. Depressive and panic disorder symptoms were under control. The patient was diagnosed 5 years before with fibromyalgia (treated with hydroxychloroquine). Family psychiatric history shows father with bipolar II disorder and brother with attention deficit and hyperactivity disorder and MDD.

After a thorough clinical examination and laboratory evaluation, we discussed with the patient the decision to start bupropion at a dosage of 150 mg. Four weeks later, the patient reported a decrease in appetite but also expressed distressing obsessive thoughts. She believed that she could accidentally drop the pills intended for her treatment and was concerned that her children might have swallowed them. These constant obsessive ideas were accompanied by compulsions of checking and intensive counting of the medication and inspection of all the corners of her house. The patient was extremely anxious and reported being unable to think and was constantly counting her medication pills throughout the day. The patient recognized that her thoughts and behavior were illogical, took more than 4 h of her day, and was unable to control them.

A revision of symptoms was done, and the MINI application excluded the previous presence of OCD (according to DSM‐5 criteria). OCS evaluations were done with Y‐BOCS and CGI‐S scales (Table 2). It was decided to suspend bupropion, and after 4 weeks, the patient observed a marked decrease in her OCS with a complete remission at weeks 8 and 16.

The prognosis of OCS was considered favorable. The patient acknowledged that her OCS had a pathological origin but initially attributed it to anxiety. It was only after her symptoms subsided that she was able to distinguish between them and her feelings of depression, as well as her genuine concerns for the safety of her children.

### 3.4. Case 4

A 58‐year‐old female housewife outpatient, mother of 6 children, with a diagnosis of bipolar I disorder since the age of 16, under long‐term treatment with quetiapine SR 450 mg qhs, quetiapine 400 mg qhs, lamotrigine 300 mg day, estazolam 4 mg qhs, zolpidem 10 mg qhs, propranolol 20 mg qam. She complained about weight gain and decreased libido associated with her treatment. No remarkable history of medical problems was recorded. Family psychiatric history shows two brothers with bipolar I disorder, one sister with gambling disorder, and one sister with MDD.

It is noteworthy that the patient was treated multiple times in the past with bupropion at doses up to 300 mg for episodes of depression that were not associated with obsessive thoughts. The first emergence of OCS was observed 1 year prior to case selection when bupropion/naltrexone combination was prescribed for an increased appetite and weight gain, reaching doses of 160/16 mg per day. During this treatment period, the patient encountered obsessive thoughts for the first time. These thoughts lasted for 2 months after discontinuing the medication, which was ineffective in managing her appetite.

One year later, the patient reported progressive depressive symptoms lasting for 2 weeks, and it was agreed to initiate bupropion 150 mg, as it had been successfully done in the past.

After 3 weeks of bupropion treatment, the patient reported experiencing intrusive and persistent religious thoughts related to blasphemy. She also had distressing mental images of harming or murdering her 2‐month‐old granddaughter. These thoughts were overwhelming, causing her to cry in despair. Although she struggled with constant obsessions, they were not accompanied by compulsive behaviors. The patient felt extremely anxious, more depressed, and experienced profound guilt about her thoughts. However, she recognized that these thoughts were irrational, as she is a devout religious person who loves her granddaughter.

A symptom evaluation was conducted, and the MINI was reapplied to exclude the previous diagnosis of OCD (according to DSM‐5 criteria). OCS evaluation was done with Y‐BOCS and CGI‐S (Table 2). Bupropion was suspended and after 4 weeks the patient observed a decrease in her OCS with a progressive reduction to weeks 8 and 16.

The prognosis for her OCS was considered favorable, given that they had resolved previously when bupropion was stopped.

## 4. Discussion

**Figure 1:**
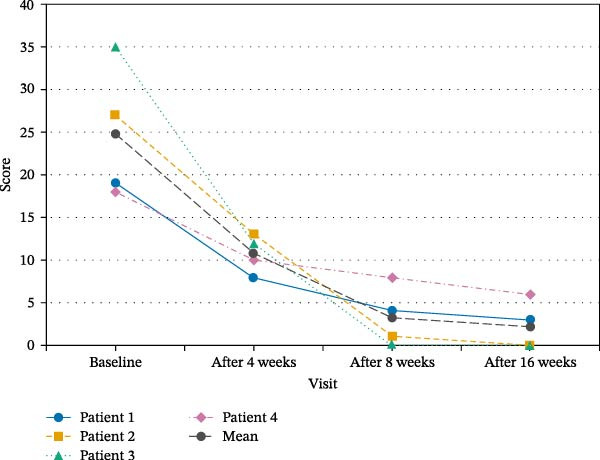
YBOCS scores. Changes in YBOCS scores from baseline and weeks 4–16 after bupropion discontinuation.

**Figure 2:**
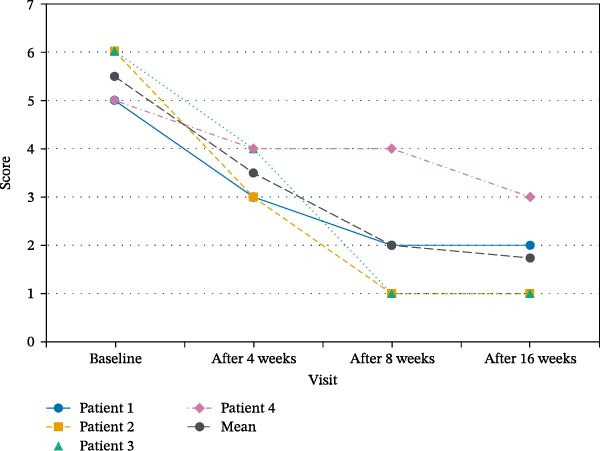
CGI‐S scores. Changes in CGI‐S scores from baseline and weeks 4–16 after bupropion discontinuation.

In all four cases, patients reported that the use of bupropion was temporally related to OCS, which gradually became more severe and disabling, and the symptoms completely resolved after it was discontinued, with observations extended for over 16 weeks. In two patients, fluvoxamine was prescribed for the management of OCS. Clinical improvement occurred following both initiation of anti‐obsessional treatment and the discontinuation of bupropion. Accordingly, the observed symptom improvement represents a confounded dechallenge, and a direct causal attribution to bupropion withdrawal alone cannot be established. We observed a gradual decrease in OCS on the YBOCS scale and CGI‐S until all patients no longer reported clinically significant symptoms (Table 2 and Figures 1 and 2) [15].

There is limited literature on OCS developing after the use of bupropion, while a dopaminergic mechanism can be proposed. Our case series does not allow causal inference, and interpretation must remain speculative. Although classical SSRIs and SNRIs do not have significant direct effects on dopamine at therapeutic doses, they can indirectly influence dopamine activity. This occurs through their modulation of serotonin and their impact on the clearance of dopamine mediated by NE, particularly in the prefrontal regions where the NET is involved in dopamine reuptake [16]. This complexity underscores how serotonergic, noradrenergic, and dopaminergic systems interact with one another in clinically significant ways.

Although bupropion’s inhibition DAT and NET suggest a possible pathway for altered dopaminergic neurotransmission, the anticholinergic–nicotinic effects and the indirect influence of serotonergic agents on dopamine levels provide alternative and potentially interacting mechanisms that may contribute to the development or modulation of OCS. As a result, it is important to consider multiple neural pathways, including the balance between dopaminergic and serotonergic systems, as well as cholinergic modulation within cortico‐striatal‐thalamo‐cortical networks linked to obsessive‐compulsive phenomena, rather than assuming a single causal mechanism [17]. Future prospective studies are necessary to clarify these pathways and their clinical significance.

In the cases presented, previous treatments with SSRIs and SNRIs were not temporally associated with the onset of OCS; nevertheless, this does not exclude possible serotonergic and noradrenergic modulation that may indirectly influence dopaminergic neurotransmission with frontostriatal circuits. Additionally, bupropion’s antagonism of nicotinic acetylcholine receptors may also play a role in the development of OCS [18].

Research has established a strong relationship between OCD and MDD. Studies have reported that intrusive thoughts are common in depression, and the severity of depression is related to higher levels of intrusive thoughts [19]. Furthermore, negative affective moods are present in both OCD and depression [20]. The use of psychotropic drugs has been linked to the development of OCS. Yıldızhan et al. [21] reported a case of ruminative ideas emerging after using high doses of venlafaxine, while Khullar et al. [22] reported a worsening of OCS with quetiapine. Case 4 received long‐term treatment with stable doses of quetiapine while also being treated with bupropion. In this case, we did not observe a worsening in symptom severity.

Bupropion is primarily metabolized by the enzyme CYP2B6 and has a clinical inhibitory effect on CYP2D6 [8]. In contrast, naltrexone is converted to 6‐β‐naltrexol through non‐CYP pathways [23]. Therefore, there is no strong evidence indicating that naltrexone causes an increase in bupropion plasma concentrations due to a direct pharmacokinetic interaction.

Research has shown mixed results regarding the use of bupropion and naltrexone for treating OCD. While some clinical trials suggested that naltrexone could serve as an augmentation treatment, other case reports indicated that it might exacerbate obsessive thoughts [24, 25]. Thus, it is unknown the effect between the two drugs in the case.

Dopamine seems to have a role in the psychophysiology of OCD. In animal models, it was suggested that OCD‐like behaviors are regulated by two distinct dopaminergic neural pathways projecting from the substantia nigra. Dopaminergic inputs from the substantia nigra facilitate OCD‐like repetitive behaviors in mice. While the activity of the substantia nigra‐ventromedial striatum pathway promotes self‐grooming behaviors (observed as a stereotyped sequence of orofacial and body‐care motor patterns) via D1 receptors, the activity of the substantia nigra‐orbitofrontal cortex pathway suppresses self‐grooming behaviors via D2 receptors [26].

To support the idea that increased dopamine levels are related to OCS in humans, studies have shown that dopamine‐releasing substances, such as amphetamine and its derivatives (like methylphenidate), can lead to compulsive behaviors in children diagnosed with ADHD. Additionally, cocaine and methamphetamine users have exhibited stereotyped movements and OCS as a result of increased dopamine [27]. On the other hand, a reduction in dopamine turnover improves OCD as shown by the effect of antipsychotics added to SSRIs in refractory groups of patients. It was published in a group of patients with OCD that some of them deteriorated in OCS when receiving bupropion [13]. Authors speculate that an increase in the availability of dopamine by bupropion could be related to this deterioration.

In the limitations of our study, it is important to note that all patients were receiving multiple pharmacological treatments for depressive symptoms. Specifically, fluvoxamine appeared to reduce OCS in two of the patients. We also identified a temporal discrepancy between the onset of OCS and the initiation of bupropion therapy, which varied between 3 weeks and 4 months. This variation complicates the understanding of the relationship between the mechanisms by which bupropion may induce OCS and the temporal association between the two events. Furthermore, the cases involved diverse patient populations, three with MDD (and two with TRD) and one with bipolar I disorder. These factors complicate the identification of a uniform neurobiological mechanism linking bupropion to the development of OCS in this cohort.

## 5. Conclusion

Bupropion may be associated with the emergence of clinically significant OCS in patients with mood disorders, typically after a delay of several weeks. In these cases, OCS improved after bupropion discontinuation, with remission observed during follow‐up.

## Funding

No funding was received for this manuscript.

## Conflicts of Interest

The authors declare no conflicts of interest.

## Supporting Information

Additional supporting information can be found online in the Supporting Information section.

## Supporting information


**Supporting Information** We provide the CARE checklist of information indicating the lines where we reported each item on the manuscript.

## Data Availability

The data that support the findings of this study are available from the corresponding author upon reasonable request.
