# Supplementary material for: Obsessive‐Compulsive Symptoms Associated With Bupropion Treatment: A Case Series
Source: Case Rep Psychiatry. 2026 Jun 11;2026:8097103. doi: 10.1155/crps/8097103 (PMC13255149; doi:10.1155/crps/8097103)
Supplement: Supplementary file 1 — Supporting Information We provide the CARE checklist of information indicating the lines where we reported each item on the manuscript. [file CRPS-2026-8097103-s001.pdf]

CARE Checklist of information to include when writing a case report

CARE case report guidelines

(cc) BY-NC-ND

Topic	Item	Checklist item description	Reported on Line
Title	1	The diagnosis or intervention of primary focus followed by the words "case report"	Line 1
Key Words	2	2 to 5 key words that identify diagnoses or interventions in this case report, including "case report"	Line 73
Abstract (no references)	3a	Introduction: What is unique about this case and what does it add to the scientific literature?	Line 50
	3b	Main symptoms and/or important clinical findings	Line 56-60
	3c	The main diagnoses, therapeutic interventions, and outcomes	Line 60-63
	3d	Conclusion—What is the main "take-away" lesson(s) from this case?	Line 64-67
Introduction	4	One or two paragraphs summarizing why this case is unique (**may include references**)	Line 95
Patient Information	5a	De-identified patient specific information.	Line 130, 173, 209, 241
	5b	Primary concerns and symptoms of the patient.	Line 131, 174, 212, 245
	5c	Medical, family, and psycho-social history including relevant genetic information	Line 134, 175, 214, 246
	5d	Relevant past interventions with outcomes	Line 140, 180, 211, 243
Clinical Findings	6	Describe significant physical examination (PE) and important clinical findings.	Line 154, 188, 228, 258
Timeline	7	Historical and current information from this episode of care organized as a timeline	Line 146, 182, 217, 251
Diagnostic Assessment	8a	Diagnostic testing (such as PE, laboratory testing, imaging, surveys).	Line 154, 188, 228, 258
	8b	Diagnostic challenges (such as access to testing, financial, or cultural)	
	8c	Diagnosis (including other diagnoses considered)	Line 137, 189, 210, 242
	8d	Prognosis (such as staging in oncology) where applicable	Line 167, 203, 233, 272
Therapeutic Intervention	9a	Types of therapeutic intervention (such as pharmacologic, surgical, preventive, self-care)	Line 158, 191, 218, 266
	9b	Administration of therapeutic intervention (such as dosage, strength, duration)	Line 158, 191, 218, 266
	9c	Changes in therapeutic intervention (with rationale)	Line 160, 196, 230, 261
Follow-up and Outcomes	10a	Clinician and patient-assessed outcomes (if available)	Line 161, 197, 231, 261
	10b	Important follow-up diagnostic and other test results	
	10c	Intervention adherence and tolerability (How was this assessed?)	
	10d	Adverse and unanticipated events	Line 150, 183, 219, 251
Discussion	11a	A scientific discussion of the strengths AND limitations associated with this case report	Line 312
	11b	Discussion of the relevant medical literature **with references**.	Line 288
	11c	The scientific rationale for any conclusions (including assessment of possible causes)	Line 279
	11d	The primary "take-away" lessons of this case report (without references) in a one paragraph conclusion	Line 322
Patient Perspective	12	The patient should share their perspective in one to two paragraphs on the treatment(s) they received	Line 164, 200, 233, 272
Informed Consent	13	Did the patient give informed consent? Please provide if requested	Yes ☒ No ☐
